# Attitudes, perceptions, behaviours, and concerns of patients listed for kidney transplantation during the COVID-19 pandemic

**DOI:** 10.1371/journal.pone.0358328

**Published:** 2026-09-15

**Authors:** Nimet Aktas, Alparslan Ersoy, Aysegul Oruc, Hatice Ortac

**Affiliations:** 1 Department of Nephrology, University of Health Sciences, Bursa Yuksek Ihtisas Education and Research Hospital, Bursa, Turkiye; 2 Department of Nephrology, Faculty of Medicine, Bursa Uludag University, Bursa, Turkiye; 3 Department of Biostatistics, Institute of Health Sciences, Bursa Uludag University, Bursa, Turkiye; Saint-Joseph University of Beirut, LEBANON

## Abstract

**Background:**

The COVID-19 pandemic caused major disruptions in solid organ transplantation worldwide, particularly in kidney transplantation. Patients on deceased-donor kidney transplant waiting lists represent a vulnerable population due to ongoing dialysis requirements and concerns regarding immunosuppression and infection risk.

**Objective:**

This study aimed to evaluate the attitudes, behaviors, and concerns of patients on the deceased-donor kidney transplant waiting list regarding kidney transplantation during the second wave of the COVID-19 pandemic.

**Methods:**

A cross-sectional, telephone-based survey was conducted between October and December 2020 at one tertiary care centre in Turkiye. The survey assessed adherence to preventive measures, COVID-19 exposure history, perceptions of renal replacement therapy modalities, attitudes toward kidney transplantation, and transplantation-related concerns during the pandemic. Demographic and clinical data were collected, and responses were analyzed using appropriate statistical methods.

**Results:**

A total of 188 transplant candidates (mean age 47.3 ± 13.1 years; 56.4% male) were included. Adherence to preventive measures was reported by 99.5% of participants, and 80.9% followed quarantine rules. Although 76% of participants preferred kidney transplantation before the pandemic, this rate decreased to 55.3% during the pandemic. Despite this decline, 86.7% of participants stated that they would accept a deceased-donor kidney transplant if offered during the pandemic. Only 38.3% reported concerns about undergoing transplantation, with the most common concerns being infection risk related to immunosuppressive therapy, hospital-acquired COVID-19, and donor-related transmission. Shorter renal replacement therapy duration and preemptive transplant status were associated with more favorable attitudes toward transplantation during the pandemic.

**Conclusions:**

Despite pandemic-related uncertainties and concerns, most patients on the deceased-donor kidney transplant waiting list demonstrated a willingness to proceed with kidney transplantation and showed high adherence to preventive measures. These findings highlight the importance of patient education, infection-control strategies, and transparent communication to ensure the sustainability of kidney transplant programs during current and future pandemics.

## Introduction

The global pandemic of the novel coronavirus (SARS-CoV-2) has precipitated a substantial reorganization of healthcare systems worldwide. This has encompassed the reallocation of hospital resources to the management of cases of the virus, the limitation of intensive care unit capacity, an elevated risk of nosocomial transmission, and the implementation of restrictive public health measures. Consequently, a significant number of elective medical procedures were postponed, and a substantial decline was evident in various healthcare services, particularly in solid organ transplantation (SOT) activities [1]. Kidney transplantation (TX) continues to be the most efficacious therapeutic modality for patients with end-stage kidney disease, offering superior survival and quality of life in comparison with dialysis-based renal replacement therapies [2]. During the 2019–2020 pandemic period, there was a global decline in SOT activity of approximately 16%, with the most significant decrease being observed in kidney TX, largely due to the emergence of alternative treatment modalities [1]. Turkiye encountered analogous disruptions in kidney TX during the pandemic. According to data from the Turkish Nephrology Association, the total number of kidney transplants decreased from 3,858 in 2019 to 2,499 in 2020, corresponding to a 35% reduction [3,4].

Patients on transplant waiting lists represent a particularly vulnerable population, as most are required to continue maintenance dialysis and attend healthcare facilities regularly, thereby increasing their exposure risk during the pandemic. This risk is especially pronounced among in-center haemodialysis (HD) patients, who frequently visit hospitals and for whom strict social distancing measures within dialysis units are challenging to implement [5]. Conversely, concerns regarding the potential adverse effects of post-transplant immunosuppressive therapy on infection susceptibility introduced substantial uncertainty into clinical decision-making processes related to kidney TX. The aforementioned challenges had repercussions for both patients awaiting TX and healthcare professionals involved in the provision of transplant care [1,6,7].

Despite the extensive research conducted on the impact of the pandemic on TX activity and recipient outcomes, there is a paucity of literature on the perception of the balance between remaining on dialysis and accepting a deceased-donor transplant among kidney transplant candidates themselves. The evidence regarding their willingness to undergo TX, TX-related concerns, treatment preferences, and adherence to preventive measures during the pre-vaccination period remains limited.

In light of the considerable uncertainty experienced during the period of the pandemic of the novel virus known as SARS-CoV-2, insights gained from this period may assist in the formulation of healthcare policies and preparedness strategies for future pandemics. The present study therefore sought to assess the attitudes of patients on the deceased donor kidney transplant waiting list towards the pandemic, their behaviours related to the virus, and their perceptions and concerns regarding kidney transplants during the second wave of the pandemic.

## Materials and metods

### Study design and setting

This cross-sectional survey study was conducted among individuals on the deceased-donor kidney transplant waiting list at one tertiary care centre in Turkiye between 1 October and 31 December 2020. This period corresponded to the second wave of the pandemic, during which the vaccination programme had not yet been initiated.

### Study population and eligibility criteria

The study population comprised adults (aged 18 years and over) who had completed the required evaluation and documentation and were registered on the deceased-donor kidney transplant waiting list at the centre. Individuals younger than 18 years of age and those whose evaluation for deceased-donor kidney TX was still ongoing or whose required documentation for waitlist registration had not yet been completed were excluded from the study. A total of 210 eligible adult kidney transplant candidates were contacted by telephone. In the event that the initial call was not answered, a maximum of three attempts to make contact were made. Seven candidates could not be contacted after three attempts, and 15 declined to participate in the study. Consequently, the final analysis comprised 188 participants.

### Survey development and content

An anonymous survey was developed by an expert committee comprising three nephrologists with expertise in kidney TX to assess the attitudes, behaviours and concerns of waitlisted kidney transplant candidates regarding kidney TX during the pandemic caused by the severe acute respiratory syndrome coronavirus 2 (SARS-CoV-2) (S1 Table). The administration of the survey was conducted via telephone in order to mitigate the risk of SARS-CoV-2 transmission during the pandemic.

Initially, demographic data were recorded during the interviews. Subsequently, the participants were invited to respond to the questions posed in the surveys.

The questionnaire consisted of yes/no questions (Q1, Q2, Q4, Q6–Q11, and Q14–Q18) and content (Q3, Q5, Q12, Q13, and Q19). The survey assessed adherence to COVID-19 preventive measures (Q1–Q5, Q10, and Q11); history of COVID-19 positivity among participants, family members, and dialysis staff, as well as contact history in the event of a positive case (Q6–Q9); perceptions of renal replacement therapy modalities (RRT) during the pandemic (Q12 and Q13); attitudes toward kidney TX (Q14–Q17); and concerns regarding kidney TX during the pandemic (Q18 and Q19). The complete questionnaire is provided in S1 Table.

### Data collection and telephone interview procedure

Telephone interviews were conducted in Turkish, the native language of the participants, using a standardised interview procedure administered by two certified transplant coordinators. The telephone number(s) recorded in the waiting-list records were used to contact eligible candidates. The interviews were conducted by two certified transplant coordinators, with calls being conducted using the speakerphone function. This allowed the second coordinator to observe the interview and serve as an independent witness during the verbal informed consent process.

### Ethical considerations

Ethical approval (IRB approval number: 2011-KAEK-26) was obtained from the Uludag University Faculty of Medicine Clinical Research Ethics Committee. Due to the risk of transmission of the SARS-CoV-2 virus, it was not possible to obtain written informed consent during the pandemic. Prior to their involvement in the survey, verbal informed consent was obtained from all participants. The verbal informed consent statement and the procedure for obtaining consent were approved by the IRB. The data were recorded anonymously. The phrase “All study procedures were conducted in accordance with the ethical standards of the relevant ethics committee and the Declaration of Helsinki.”

### Statistical analysis

Statistical analyses were performed with SPSS software (SPSS: An IBM Company, version 25.0, IBM Corporation, Armonk, NY, USA). Shapiro Wilk test was used to evaluate the suitability of variables for normal distribution. The numerical and categorical variables were expressed as the mean ± standard deviation or median (minimum: maximum) and ratios (%), respectively. Comparison of groups was carried out by using Mann-Whitney U, Kruskal Wallis, Pearson chi-square, Fisher-Freeman-Halton and Fisher’s exact tests. A p value <0.05 was considered statistically significant.

## Results

Of the 210 eligible kidney transplant candidates who were contacted by telephone, 188 were included in the final analysis. Seven patients could not be reached, and 15 patients declined to participate in the survey. The overall survey response rate was 89.5%. The mean age of the participants was 47.3 ± 13.1 years, and 106 (56.4%) were male. The demographic and clinical characteristics of the study population are presented in Table 1.

**Table 1:** Demographic and clinical characteristics of the participants.

Parameter	Value
**Age** (years)	47.3 ± 13.1
**Sex, n (%)**	
Female	82 (43.6)
Male	106 (56.4)
**Educational level, n (%)**	
Illiterate	2 (1.1)
Primary school	77 (41.0)
Secondary school	34 (18.1)
High school	64 (34.0)
University	11 (5.8)
**Marital status, n (%)**	
Single	53 (28.2)
Married	125 (66.5)
Divorced/Widowed	10 (5.3)
**Smoking status, n (%)**	
Current smoker	24 (12.8)
Former smoker	41 (21.8)
Never smoker	123 (65.4)
**Presence of chronic disease, n (%)**	
No	50 (26.7)
Cardiovascular disease	3 (1.6)
Diabetes mellitus	3 (1.6)
Hypertension	107 (56.9)
Cancer	1 (0.5)
Other	24 (12.7)
**History of kidney transplantation, n (%)**	24 (12.8)
**Donor type of previous transplantation, n (%)**	
Living	10 (41.7)
Deceased	14 (58.3)
**Dialysis modality, n (%)**	
No dialysis (preemptive)	26 (13.8)
HD	134 (71.3)
PD	28 (14.9)
**HD modality, n (%)**	
Home HD	9 (6.7)
Center HD	125 (93.3)
**PD modality, n (%)**	
CAPD	17 (60.7)
APD	11 (39.3)
**Number of HD sessions per week, n (%)**	
2 sessions	16 (12.0)
3 sessions	118 (88.0)
**Dialysis vintage (months)**	16 ± 32.7
**Duration on the waiting list (months)**	15.3 ± 27.4
**Number of previous calls for transplantation**	0.6 ± 1.2

Abbreviations: HD, hemodialysis; PD, peritoneal dialysis; CAPD, continuous ambulatory peritoneal dialysis; APD, automated peritoneal dialysis. Data are presented as mean ± standard deviation or n (%).

The modality of RRT for the candidates was HD in 134 (71.3%) participants and peritoneal dialysis (PD) in 28 (14.9%) participants. Twenty-six (13.8%) candidates were not undergoing dialysis at the time. Twenty-four candidates (representing 12.8% of the study population and including 14 from deceased-donor transplants) had previously undergone kidney TX. The duration of the RRT was found to be 16 ± 32.7 months, and the duration on the waiting list was 15.3 ± 27.4 months. Of the 134 patients included in the study, 118 (88%) were enrolled in the thrice-weekly HD programme. During the study period, it was determined that there were no changes in the treatment programmes of HD patients due to the pandemic.

Responses to survey questions on pandemic-related preventive measures and exposure to the virus, along with related attitudes, are presented in Table 2 below.

**Table 2:** Responses to survey questions on pandemic-related preventive measures and regarding COVID-19 exposure, related attitudes.

A. Responses to Survey Questions on Pandemic-Related Preventive Measures			
**Question**	**YES**	**NO**	
Q1: Did you comply with social isolation and personal hygiene measures during the pandemic period?(n,%)	187(%99.5)	1 (%0.5)	
Q2: Did you need to receive assistance to meet your daily needs during the pandemic period?(n,%)	52 (%27.7)	136(%72.3)	
Q4: Did your mode of transportation to your dialysis center change during the pandemic? *(n,%)	14 (%11.2)	111(%88.8)	
Q10: Did you self-quarantine during the pandemic? (n,%)	152(%80.9)	36 (%19.1)	
Q11: During the pandemic, were you informed about COVID-19 and the preventive measures to be taken by the healthcare professionals responsible for your treatment at the dialysis unit? (n,%)	165(%87.8)	23 (%12.2)	
Q3: What type of transportation did you use to access your dialysis center? (n,%)	Public transportation16(%8.5)	Private vehicle 58 (%30.9)	Shuttle service 114(%60.6)
**B. Participants’ Responses to Questionnaire Items Regarding COVID-19 Exposure and Related Attitudes**			
**Question**	**YES**	**NO**	
Q6: Was COVID-19 positivity detected in you, your family members,or your neighbors during the pandemic?(n,%)	41 (%21.8)	147(%78.2)	
Q7: If COVID-19 positivity was detected in your family members or neighbors, did you have contact with these individuals? **(n,%)	20 (%48.8)	21 (%51.2)	
Q8: During the pandemic, were there any dialysis staff members at your dialysis center who tested positive for COVID-19? ***(n,%)	46 (%35.7)	83 (%64.3)	
Q9: If COVID-19 positivity was detected at your dialysis center, did you have contact with these individuals? ****(n,%)	25 (%54.3)	21 (%45.7)	

Abbreviation: Q: Question. Data are presented as n(%). *Only 125 volunteers receiving in-center hemodialysis treatment were included. **Q 7 was administered to a total of 41 participants who answered “Yes” to Q 6. *** Q 8 was administered to a total of 129 participants, including 125 receiving in-center hemodialysis and 4 peritoneal dialysis patients who attended the center during the survey period. ****Q 9 was administered to a total of 46 participants who answered“Yes.”

In the cohort of centre HD patients, only 14 out of 125 (11.2%) required a change in their designated transport vehicle. In the present study, it was found that 85.7% of the participants ceased to utilise the transportation service provided by the centre, opting instead to arrange their own transportation by private vehicle. In contrast, 14.3% of the participants reported a transition from public transportation to private vehicle use. With the exception of the in-centre HD patients, the remaining 63 participants (not on RRT, n = 26; PD, n = 28; and home HD, n = 9) utilised private vehicles (62.4%) and public transport (37.6%) for transportation to their appointments.

However, the frequency of hospital visits among participants other than those receiving in-center HD demonstrated marked variability. From the onset of the pandemic until the conclusion of the study period, a notably high proportion of participants who were not receiving RRT or were on home-based dialysis modalities had no hospital visits (93.7%, n = 59). Participants demonstrated a commendable success rate in implementing prevention strategies, with a 99.5% compliance rate. Furthermore, 80.9% of participants reported adhering to quarantine regulations. A total of 52 candidates (27.7%) on the waiting list reported receiving assistance for their needs during the pandemic (Table 2). From the onset of the pandemic until the survey period, the prevalence of the novel coronavirus among participants’ family members and social contacts was 21.8%, whereas the corresponding rate among dialysis centre staff was 35.7%. While 51.2% of participants reported that they were able to avoid contact with cases of the virus in their family and social environment, only 45.7% of participants reported that they had managed to avoid exposure to dialysis staff who had tested positive for the novel virus (Table 2).

Participants’ perspectives on RRT, as well as their evaluations and opinions on TX activity before and during the pandemic, are presented in Table 3.

**Table 3:** Participants’ perspectives on renal replacement therapy and opinions on kidney transplantation before and during the COVID-19 pandemic.

A. Participants’ Perspectives on Renal Replacement Therapies				
**Question**	**HD**	**PD**	**Home HD**	**Kidney TX**
Q12: Which treatment modality do you think was most advantageous for you during the pandemic?n (%)	38 (20.2%)	16 (8.5%)	30 (16%)	104 (55.3%)
Q13: If there had been no pandemic, which treatment modality would you have preferred?n (%)	28 (14.9%)	5 (2.7%)	12 (6.4%)	143 (76%)
**B. Participants’ Opinions on Transplantation Activity Before and During the Pandemic**				
**Question**	**Yes, n (%)**		**No, n (%)**	
Q14: Were you called to our center for deceased-donor kidney TX before the pandemic?	54 (28.7%)		134 (71.3%)	
Q15: During the pandemic, were you called to our center for deceased donor kidney TX?	17 (9%)		171 (91%)	
Q16: If you had been called to our center for kidney TX during the pandemic, would you have agreed to undergo TX?	163 (86.7%)		25 (13.3%)	
Q17: If there had been no pandemic, and you had been called to our center for kidney TX, would you have agreed to undergo TX?	182 (96.8%)		6 (3.2%)	
Q18: Would you have been concerned if you had undergone kidney TX during the pandemic?	72 (38.3%)		116 (61.7%)	
Q19: If your answer is yes, what would be your main concerns about undergoing TX during the pandemic?*				
**Concern**				**n (%)**
Concern about acquiring COVID-19 infection in the hospital				34 (47.2%)
Concern about COVID-19 transmission from the deceased donor				27 (37.5%)
Concern about acquiring COVID-19 infection during the surgical procedure				26 (36%)
Concern about disruption of hospital follow-up visits				26 (36%)
Risk of COVID-19 infection due to the use of immunosuppressive medications required for TX				37 (51.4%)

Abbreviations: HD, hemodialysis; PD, peritoneal dialysis; TX, transplantation; Q, Question. Data are presented as n (%).

*Q 19 was administered to a total of 72 participants who answered “Yes” to Q 18, and multiple responses were allowed for each participant.

With regard to perceptions about TX, 143 (76%) participants stated that they would have preferred TX if the pandemic had not occurred, while 104 (55.3%) participants stated that TX was the most advantageous modality during the pandemic. A total of 54 candidates (28.7%) on the waiting list had been called for a deceased donor before the pandemic. During the pandemic, 21 candidates (11.2%) received deceased-donor kidney transplant offers, and all accepted the offers, 163 candidates (86.7%) stated that they would accept a transplant if they were called for a deceased donor. The acceptance rate (96.8%) exhibited minimal variation among candidates prior to the onset of the pandemic. With regard to concerns regarding the undertaking of transplant surgery during the pandemic, a total of 72 participants (38.3%) reported feelings of concern. The primary concerns pertained to the transmission of the virus within hospitals (47.2%), the transmission of the virus from deceased donors (37.5%), the transmission of the virus during surgical procedures (36%), the disruption of hospital controls (36%), and the transmission of the virus due to immunosuppressive medications (51.4%).

Treatment preferences and transplant-related attitudes during the pandemic were further evaluated in relation to RRT duration, time on the deceased-donor kidney transplant waiting list, and RRT modality, as presented in S2 Table. As anticipated, subjects with protracted periods of RRT and extended times on the transplant waiting list exhibited a heightened probability of being invited for kidney TX both prior to and during the pandemic. Conversely, participants who reported a willingness to accept kidney TX during the pandemic had statistically significantly shorter RRT durations and shorter durations on the deceased-donor kidney transplant waiting list.

Participants with shorter RRT durations and preemptive kidney transplant candidates more frequently reported that kidney TX was a more advantageous treatment option during the pandemic. In addition, compared with participants receiving alternative treatment modalities, preemptive candidates on the deceased-donor kidney transplant waiting list were more likely to perceive kidney TX as a beneficial treatment option and to report more positive attitudes towards kidney TX during the pandemic (S2 Table).

## Discussion

The advent of the pandemic precipitated a comprehensive reassessment of established nephrology clinical practices, a consequence of the considerable disruption to transplant programmes that ensued. In this regard, we encountered challenges in the TX practice. As is the case in numerous countries, Turkiye has maintained a cautious approach to kidney TX, adhering to established guidelines. Despite the concerns expressed by TX candidates regarding the pandemic, the willingness of these individuals to undergo TX was noteworthy in the present study.

The present study examined the acceptance rate of a deceased-donor kidney transplant offer during the pandemic. The analyses revealed that 86.7% of participants accepted the offer, while 13.3% reported that they would decline in case of a deceased organ offer. In the non-pandemic period, this rate increased to 96.8%. This discrepancy may be indicative of an elevated perception of risk associated with the infection among participants. Indeed, it has been reported that 21% of kidneys accepted in the United States in 2020 were ultimately not transplanted due to the inability to identify a suitable recipient [8]. Notably, all 21 deceased-donor kidney offers extended to study participants at our institution were successfully accepted.

This finding is consistent with evidence from different healthcare systems showing that patients on waiting lists maintained a strong preference for kidney TX despite pandemic-related risks. Concerns regarding infection with the SARS-CoV-2 virus and the impact of post-transplant immunosuppression were likely given due consideration when making a risk-benefit assessment, which suggested that the benefits of TX outweighed the risks when compared with the substantial burden of prolonged dialysis and the established survival and quality-of-life benefits of TX [9–11].

When changes in preferences regarding RRT modalities were examined, the preference for kidney TX decreased from 76% in the pre-pandemic period to 55.3% during the pandemic. During this period, the following factors influenced patients’ perceptions of risk: limited knowledge regarding SARS-CoV-2 transmission; the absence of effective vaccines; concerns about the effects of immunosuppressive therapy; and reports of poor outcomes among infected transplant recipients [12–14]. Moreover, the pervasive suspension or substantial reduction of TX programmes on a global scale may have served to reinforce the perception that TX constituted a higher-risk intervention under pandemic conditions [15,16].

During the pandemic, 38.3% of participants indicated that they would be concerned about undergoing a kidney transplant. In accordance with the findings of preceding survey-based studies, the most prevalent concerns among our participants, in descending order of frequency, encompassed the potential consequences of post-transplant immunosuppressive therapy, the possibility of acquiring a SARS-CoV-2 infection during hospitalisation, the risk of transmission of SARS-CoV-2 from the deceased donor, the risk of perioperative infection, and concerns regarding disruptions in post-transplant follow-up care [10,11,16,17].

In the present survey, the attitudes and approaches of patients on the deceased-donor kidney transplant waiting list towards pandemic-related preventive measures were evaluated. These measures represent a critical factor for the continuation of transplant activities during this period. The finding that 99.5% of participants reported adherence to preventive measures and 80.9% reported practicing self-isolation was consistent with findings and recommendations reported in the literature [9–11,18]. Approximately one-quarter of participants required assistance, highlighting the potential importance of family and social support in maintaining preventive behaviors and continuity of care [16,19]. Despite high adherence to preventive measures, avoiding exposure remained challenging, particularly in HD settings. SARS-CoV-2 positivity among dialysis-center staff was 35.7%, and 54.3% of participants reported contact exposure. This finding is consistent with the increased infection risk reported among HD patients [5,20] and underscores the importance of robust organizational and infection-control measures in dialysis centers during pandemic conditions.

The subgroup analyses suggest that transplant-related attitudes during the pandemic were associated with RRT modality and treatment experience. All preemptive candidates were willing to accept TX if a suitable organ became available, whereas PD patients were more likely than HD patients to prefer continuing their current treatment, consistent with reports suggesting lower healthcare-related exposure concerns with home dialysis [16,21,22]. Conversely, a preceding survey revealed no disparities in acceptance of TX based on dialysis modality or preemptive status [17]. In the present study, candidates with a shorter RRT duration and shorter time on the deceased-donor waiting list were more likely to express a preference for TX and reported less concern about undergoing TX. Despite the statistically significant differences in RRT duration regarding willingness and concern, the absolute differences were minimal (approximately 1.5–2 months). This necessitates a cautious interpretation of their clinical relevance (S2 Table). These findings may be indicative of the influence of treatment experience on decision-making processes, while the universal willingness of preemptive candidates may reflect a desire to avoid the long-term burden of dialysis and an expectation of enhanced quality of life following TX.

The findings of this study also have implications for the management of transplant patients during future infectious disease outbreaks. Maintaining TX activity may remain appropriate when adequate donor screening, recipient evaluation, and institutional infection-control measures are ensured [10,11,16,17]. The provision of individualised counselling, transparent risk communication, shared decision-making, and psychosocial support may help address concerns regarding infection, immunosuppression, donor safety, and post-TX follow-up, while supporting informed decisions and continuity of TX programs [9–11,16,17].

Strengths of this study include its focus on a relatively homogeneous deceased-donor kidney transplant candidate population, high response rate, standardized interviews during the second pandemic wave, and comprehensive assessment of treatment preferences, concerns, and preventive behaviors. To our knowledge, this is the first study from Turkiye to comprehensively evaluate these attitudes among waitlisted candidates during the pre-vaccination period. Limitations include the cross-sectional, single-center design, limited generalizability and causal inference, and potential recall and social desirability bias. The survey primarily assessed stated preferences rather than actual behavior, and perceptions may have evolved with advances in scientific knowledge, vaccination, and transplant practice. Nevertheless, capturing patients’ perspectives during this uniquely uncertain pre-vaccination period remains an important strength.

## Conclusion

Despite the uncertainty of the pre-vaccination phase of the COVID-19 pandemic, most deceased-donor kidney transplant candidates remained willing to undergo TX while maintaining high adherence to preventive measures. These findings suggest that candidates weighed pandemic-related risks against the established long-term benefits of kidney TX. During future public health emergencies, continuity of transplant programs may therefore be supported by comprehensive infection-control measures, transparent communication, individualized counselling, and shared decision-making. Particularly in countries with prolonged deceased-donor kidney transplant waiting times and low TX rates, coordinated efforts among patients, transplant teams, and healthcare policymakers, together with well-organized strategies to maintain TX activity, may help prevent disruption and ensure the continuity of transplant programs during future pandemics.

## Supporting information

S1 Table Survey questions assessing attitudes toward kidney transplantation among candidates on the waiting list during the COVID-19 pandemic. (DOCX)

S2 Table Factors associated with treatment preferences and transplant-related attitudes according to renal replacement therapy characteristics during the COVID-19 pandemic. (DOCX)

S3 Data De-identified dataset underlying the findings reported in this study. (XLSX)
