# Supplementary material for: Attitudes, perceptions, behaviours, and concerns of patients listed for kidney transplantation during the COVID-19 pandemic
Source: PLoS One. 2026 Sep 15;21(9):e0358328. doi: 10.1371/journal.pone.0358328 (PMC13577374; doi:10.1371/journal.pone.0358328)
Supplement: Supplementary file 1 [file pone.0358328.s001.docx]

**S1 Table. Survey Questions Assessing Attitudes Toward Kidney Transplantation Among Candidates on the Waiting List During the COVID-19 Pandemic**

| Question No-Content | Response Options |
| --- | --- |
| 1- Did you comply with social isolation and personal hygiene measures(hand hygiene, disinfectant use, and mask wearing) during the pandemic period? | YES/NO |
| 2- Did you need to receive assistance to meet your daily needs during the pandemic period (e.g., spouse, children, neighbors, relatives)? | YES/NO |
| 3- What type of transportation did you use to access your dialysis center? | Public transportation / Private vehicle/Shuttle service |
| 4- Did your mode of transportation to your dialysis center change during the pandemic? | YES/NO |
| 5- If your mode of transportation changed, how did it change? | Public transportation / Private vehicle/Shuttle service |
| 6- Was COVID-19 positivity detected in you, your family members,or your neighbors during the pandemic? | YES/NO |
| 7- If COVID-19 positivity was detected in your family members or neighbors, did you have contact with these individuals? | YES/NO |
| 8- During the pandemic, were there any dialysis staff members at your dialysis center who tested positive for COVID-19? | YES/NO |
| 9- If COVID-19 positivity was detected at your dialysis center, did you have contact with these individuals? | YES/NO |
| 10- Did you self-quarantine during the pandemic? | YES/NO |
| 11- During the pandemic, were you informed about COVID-19 and the preventive measures to be taken by the healthcare professionals (nurses/physicians) responsible for your treatment at the dialysis unit? | YES/NO |
| 12- Which treatment modality do you think was most advantageous for you during the pandemic? | HD/PD/HomeHD/ Kidney TX |
| 13- If there had been no pandemic, which treatment modality would you have preferred? | HD/PD/HomeHD/ Kidney TX |
| 14- Were you called to our center for deceased-donor kidney transplantation before the pandemic? | YES/NO |
| 15- During the pandemic, were you called to our center for deceased donor kidney transplantation? | YES/NO |
| 16- If you had been called to our center for kidney transplantation during the pandemic, would you have agreed to undergo transplantation? | YES/NO |
| 17- If there had been no pandemic, and you had been called to our center for kidney transplantation, would you have agreed to undergo transplantation? | YES/NO |
| 18- Would you have been concerned if you had undergone kidney transplantation during the pandemic? | YES/NO |
| 19- If your answer is yes, what would be your main concerns about undergoing transplantation during the pandemic?  (Multiple responses allowed) | - Concern about acquiring COVID-19 infection in the hospital - Concern about COVID-19 transmission from the deceased-donor - Concern about acquiring COVID-19 infection during the surgical procedure - Concern about disruption of hospital follow-up visits - Risk of COVID-19 infection due to the use of immunosuppressive medications required for transplantation |
