# Supplementary material for: Attitudes, perceptions, behaviours, and concerns of patients listed for kidney transplantation during the COVID-19 pandemic
Source: PLoS One. 2026 Sep 15;21(9):e0358328. doi: 10.1371/journal.pone.0358328 (PMC13577374; doi:10.1371/journal.pone.0358328)
Supplement: Supplementary file 2 [file pone.0358328.s002.docx]

## S2 Table. Factors associated with treatment preferences and transplant-related attitudes according to renal replacement therapy characteristics during the COVID-19 pandemic

| **Survey item** | **Response** | **RRT duration (months) Median (min–max)** | **Waiting-list duration (months) Median (min–max)** | **RRT Modality** | | | **Overall comparison**  **(p values)** |
| --- | --- | --- | --- | --- | --- | --- | --- |
|  |  |  |  | **Preemptive n (%)** | **PD n (%)** | **HD n (%)** |  |
| Which treatment modality do you think was most advantageous for you during the pandemic? (Q12) | HD | 4.5 (1–180) | 3 (1–156) | 0 | 0 | 38 (28.4) | **RRT duration: 0.005ᵃ** Waiting-list duration: 0.178ᵃ **RRT Modality: <0.001ᵇ** |
|  | PD | 8.5 (0–49) | 10.5 (1–52) | 0 | 15 (53.6) | 1 (0.7) |  |
|  | Home HD | 4.5 (0–96) | 5.5 (0–158) | 2 (7.7) | 6 (21.4) | 22 (16.4) |  |
|  | Kidney TX | 2 (0–204)# | 3 (1–133) | 24 (92.3) # | 7 (25.0) | 73 (54.5) |  |
| If there had been no pandemic, which treatment modality would you have preferred? (Q13) | HD | 4 (1–180) | 3 (1–156) | 0 | 2 (7.1) | 26 (19.4) | RRT duration: 0.150ᵃ Waiting-list duration: 0.810ᵃ **RRT Modality: <0.001ᵇ** |
|  | PD | 3 (0–36) | 3 (1–12) | 1 (3.8) | 4 (14.3) | 0 |  |
|  | Home HD | 4.5 (0–144) | 3.5 (0–97) | 1 (3.8) | 3 (10.7) | 8 (6.0) |  |
|  | Kidney TX | 3 (0–204) | 4 (1–133) | 24 (92.3) # | 19 (67.9) | 100 (74.6) |  |
| Were you called to our center for deceased-donor kidney TX before the pandemic? (Q14) | Yes | 7 (0–180) | 5.5 (1–156) | 2 (7.7) | 8 (26.8) | 44 (32.8) | **RRT duration: <0.001ᶜ** **Waiting-list duration: <0.001ᶜ** **RRT Modality: 0.035ᵇ** |
|  | No | 2 (0–204) | 3 (0–133) | 24 (92.3) # | 20 (71.4) | 90 (67.2) |  |
| During the pandemic, were you called to our center for deceased donor kidney TX? (Q15) | Yes | 10 (1–180) | 20 (2–156) | 0 | 4 (14.3) | 13 (9.7) | **RRT duration: 0.003ᶜ** **Waiting-list duration: 0.006ᶜ** RRT Modality: 0.142ᵇ |
|  | No | 3 (0–204) | 3 (0–133) | 26 (100) | 24 (85.7) | 121 (90.3) |  |
| If you had been called to our center for kidney TX during the pandemic, would you have agreed to undergo TX? (Q16) | Yes | 3 (0–204) | 3 (0–156) | 26 (100) # | 18 (64.3) | 119 (88.8) | **RRT duration: 0.046ᶜ** **Waiting-list duration: 0.005ᶜ** **RRT Modality: <0.001ᵇ** |
|  | No | 5 (0–85) | 14 (1–111) | 0 | 10 (35.7) | 15 (11.2) |  |
| Would you have been concerned if you had undergone kidney TX during the pandemic? (Q18) | Yes | 4.5 (0–204) | 7 (0–156) | 8 (30.8) | 15 (53.6) | 49 (36.6) | **RRT duration: 0.025ᶜ** **Waiting-list duration: 0.001ᶜ** RRT Modality: 0.169ᵇ |
|  | No | 3 (0–132) | 3 (1–125) | 18 (69.2) | 13 (46.4) | 85 (63.4) |  |

Data are presented as median (minimum–maximum) or n (%). Abbreviations: RRT, renal replacement therapy; HD, hemodialysis; PD, peritoneal dialysis; TX, transplantation. ᵃ , Kruskal–Wallis test. ᵇ, Fisher–Freeman–Halton test. ᶜ, Mann–Whitney U test. #: the group with statistical significance
